# 3D volumetric changes and force delivery at the attachment region of different aligner materials

**DOI:** 10.1007/s00784-025-06733-3

**Published:** 2026-03-04

**Authors:** R. Taheri, C. García-Marín, M. Aizpuru-Arotzena, A. Iglesias-Linares

**Affiliations:** 1 Department of Orthodontics, School of Dentistry Complutense University of Madrid, Madrid, Spain; 2 BIOCRAN, Craniofacial Biology and Orthodontics Research Group, School of Dentistry, Complutense University of Madrid, Pl. de Ramón y Cajal, s/n, Madrid, 28040 Spain

**Keywords:** Aligner, Clear thermoplastic appliances, Thermoplastic materials, Mechanical properties, Disinsertion force, Simulated intraoral environment

## Abstract

**Objectives:**

The aim of this in vitro study was to compare the volumetric changes in the attachment regions of two aligner materials subjected to a cyclic insertion-disinsertion model, as well as the influence of a simulated intraoral environment (SIE) on the disinsertion force of the aligners.

**Materials and methods:**

Passive aligners of each material were obtained from two patients with different intraoral conditions (mild and severe crowding). Half of these aligners were immersed in SIE. Three study times were established at 35, 50, and 75 insertion disinsertion cycles. At each study time, the aligners were scanned with a Trios 3 intraoral scanner, and the STL models obtained were superimposed for comparative analysis of the volumetric change. The mean and maximum disinsertion forces were also quantified.

**Results:**

An increase in the mean force of disinsertion was observed for both aligner materials after exposure to SIE, with a statistically significant increase observed for material 1 (*p* < 0.05). Following exposure to SIE, the aligners exhibited a mean rise in maximum disinsertion force of 56.2% in contrast to their performance under ideal conditions. However, there were no statistically significant differences in volumetric changes regardless of the magnitude of crowding and exposure to SIE.

**Conclusions:**

Exposure to SIE adversely affects aligner deformation and disinsertion resistance. The PET-G based polymer demonstrated a consistently greater tendency toward volumetric change than the Polyutherane-based polymer.

**Clinical relevance:**

Intraoral aging can significantly affect aligner force and volumetric changes, reducing the predictability of planned tooth movements.

**Supplementary Information:**

The online version contains supplementary material available at 10.1007/s00784-025-06733-3.

## Introduction

The use of dental aligners has exponentially increased over the past two decades as an alternative type of treatment to fixed appliances for mild to moderate malocclusions [1, 2]. In addition, with the improvement of materials, computer-aided manufacturing processes, and the introduction of anchorage zones and attachments, the range of movement and predictability achieved have improved [3, 4].

Dental movements with aligners are based on pressure force mechanics rather than the traditional traction mechanics of conventional fixed appliances. This type of movements are achieved by shape-modeling and the use of auxiliary elements such as attachments and pressure points [1, 5]. However, the shape-modeling effect is the most used system. This is based on the progressive movement of the teeth from the initial geometric position to the expected final position through small changes in the aligner’s shape [5, 6]. Thus, the correct adjustment of the aligner on the tooth surface remains critical for the accurate transmission of the force systems and the conservation of anchorage units. The exact extent to which new aligner materials influence treatment efficacy still requires investigation [7, 8].

Most of the current aligner materials use modified polyethylene glycol terephthalate (PET-G), polypropylene, polycarbonate (PC), thermoplastic polyurethane (TPU) OR ethylene vinyl acetate [9, 10]. In addition, the thickness of the traditional thermoformed aligner materials typically varies from 0.5 to 0.8 mm, which modifies the material’s mechanical properties [11, 12]. The thickness of the aligner materials and their adaptation to the surface of the teeth have been studied depending on the manufacturing process and the temperature reached in this process [13, 14]. In addition, the polymeric structure of the materials absorbs water in the oral environment, which can lead to degradation and perturbation of their biomechanical properties [15, 16]. It has been observed that in the oral environment, polymers suffer a modification of their mechanical properties due to chewing forces and the effect of dental enzymes [17–20].

The aim of this study was to evaluate the deformation of the attachment region of the upper left first molar of two aligner materials subjected to ideal conditions (non-exposure to simulated intraoral environment) and simulated intraoral environment (SIE) after a cyclic insertion-disinsertion wear model. The secondary aim was to study the progressive changes in the maximum force and mean force of disinsertion of the aligner from a printed model during a standardized cyclic insertion-disinsertion wear protocol.

## Materials and methods

### Study design, intraoral conditions, and aligners material

**Fig. 1:**
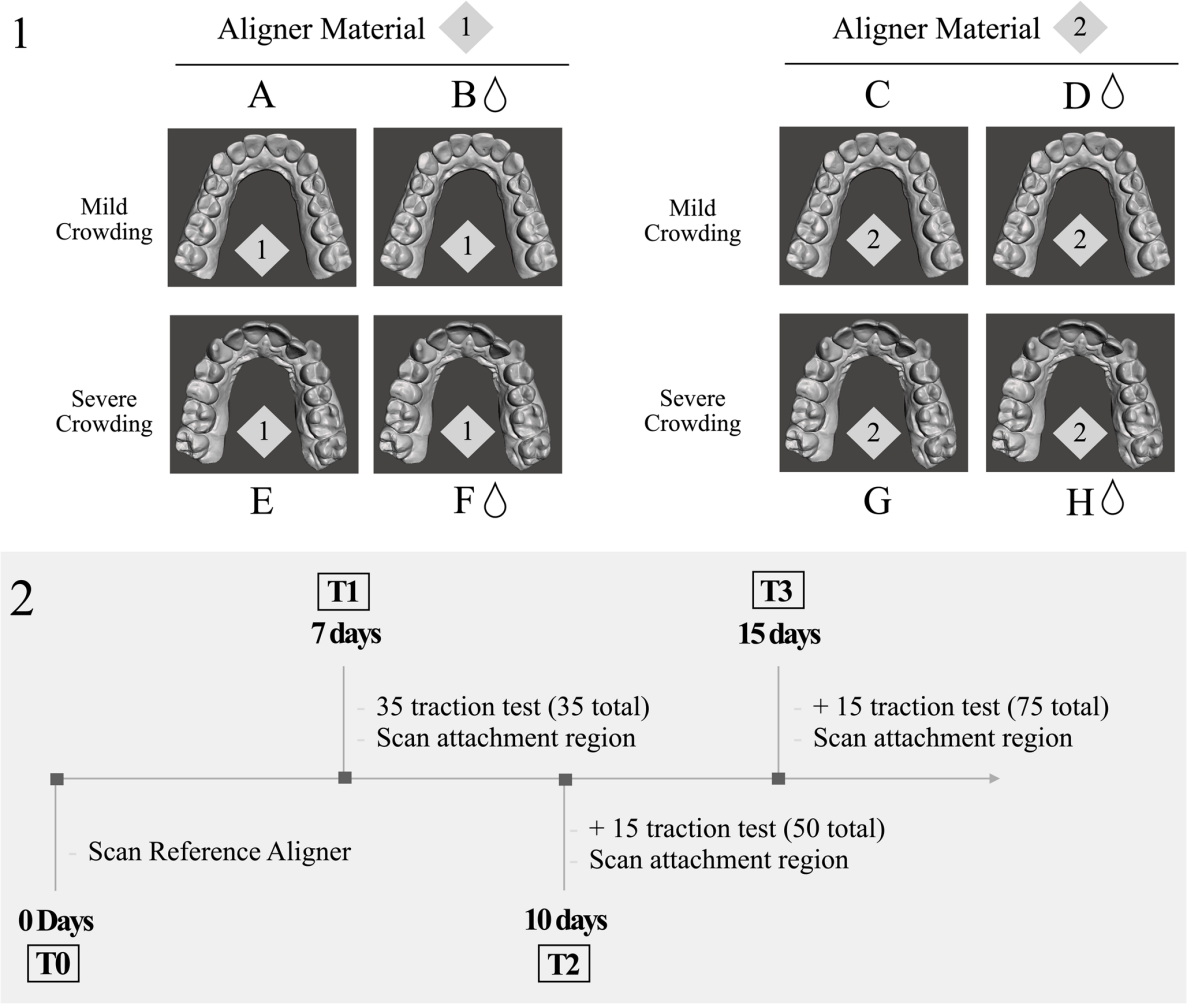
Study design; (1.1) Group allocation of the aligners depending on exposure to simulated intraoral environment, crowding and aligner material. (1.2) Observation times of the study

An experimental in vitro study was designed. Digital replicas were obtained from two patients with different intraoral conditions (mild and severe crowding) with a Trios 3 intraoral scanner (3 Shape A/S, Copenhagen, Denmark). Passive thermoformed aligners (with no active force applied) were generated from two different materials: a Polyutherane-based polymer named SmartTrack^®^ by Align Technology^®^ (Invisalign^®^ aligners, San Jose, CA, USA) (Aligner material 1) and a PET-G-based polymer by Quick Smile aligners (Quick Smile^®^, S.L, Madrid, Spain) (Aligner material 2) for each intraoral condition. A total of 4 passive aligners were used for each intraoral condition for each material. (Fig. 1−1.1).

### Specimens Preparation

**Fig. 2:**
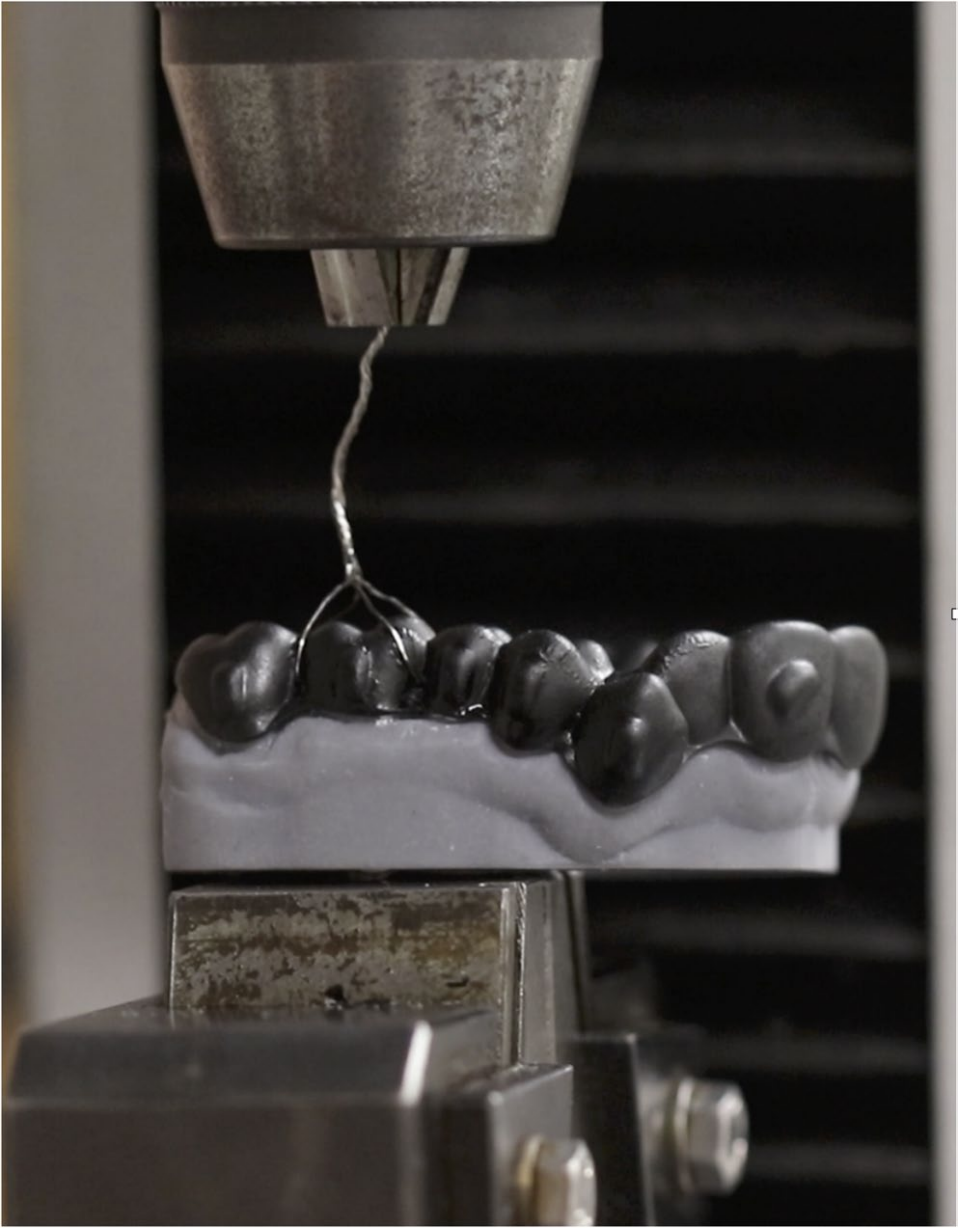
Universal testing machine. Aligner attached to printed model ready for cyclic insertion-disinsertion wear model

In the design of the passive aligners included in the study, vertical rectangular attachments were positioned on all teeth from the canine to the upper second molars. The size of the attachments in the molars were ~ 5 × 2 × 1 mm (10 mm^3^). Resin models were printed from the original STL files before the start of orthodontic treatment with a 3D printer (Pro S-Sprint Ray^®^, Los Angeles CA, USA). The resin used was Sprint Ray Die and Model 2 Gy (SRI-02020011). The attachments were bonded to the printed models using the attachment templates provided by both aligner systems with Assure^®^ Plus All Surface Bonding Resin (Reliance Orthodontic Products Inc., Itasca, IL, USA) and Tetric EvoCeram^®^ Bulk Fill composite (Ivoclar Vivadent AG, Liechtenstein). (Fig. 2; Supplementary file 1)

### Mechanical cyclical experiments: maximum/mean disinsertion force (N) calculation

The passive aligners were subjected to the insertion/disinsertion cycles. The experiments were carried out using a universal testing machine Venco s.a. (serial number H5000M-794//S/C and certificate number 1710/60). The printed models were attached to the base of the traction machine with a screw system. In addition, four anchorage points were prepared on the vestibular and palatal surface of the upper left first molar of each dental aligner. Additionally, a.008” diameter braided steel wire (Rayher 4079000) was inserted in the perforations (anchorage points) and used as a connection point to the moving end of the machine for traction. A total of 75 cycles were performed in each aligner for 15 days. This was calculated based on the minimum number of times the patient inserts and removes the aligner from their mouth, estimated to be an average of 5 times daily. Four observation times (T0, T1, T2, and T3) were established during the experiment. Consequently, the T0 value corresponded to test number 0, the beginning of the study. T1 corresponded to 7 days of use of the aligner (35 insertion/disinsertion cycles), T2 corresponded to 10 days (50 insertion/disinsertion cycles), and T3 corresponded to 15 days (75 insertion/disinsertion cycles). (Figs. 1−1.2) The aligners were subjected to insertion/disinsertion cycles under ideal and SIE conditions. For the SIE, the artificial saliva was composed of CaCl2, NaH2PO4, KCL, NACL, Na₂S, and H2NCONH2, corresponding to the neutral Fusayama–Meyer formulation (NaCl 0.4 g/L, KCl 0.4 g/L, CaCl₂·2H₂O 0.906 g/L, NaH₂PO₄·2H₂O 0.69 g/L, Na₂S·9H₂O 0.005 g/L, urea 1.0 g/L). The pH was adjusted to 6.8–6.9 to simulate neutral intraoral conditions, and the solution was maintained at 37 °C during the 15-day immersion period. These were removed from the SIE on days 7 (T1), 10 (T2) and 15 (T3). They were tested with the insertion/disinsertion cycles and scanned to obtain an STL file before they were submerged again in SIE. (Fig. 1−1.2)

During the insertion/disinsertion cycles, two main outcomes were recorded: (i) the *maximum force* implied to disinsert the aligner and (ii) the *mean force of disinsertion* of each aligner after 75 cycles. (Fig. 2; Supplementary file 1).

### Digital superimposition and deformation measurement (mm^3^) at the attachment region of the first left molar

**Fig. 3:**
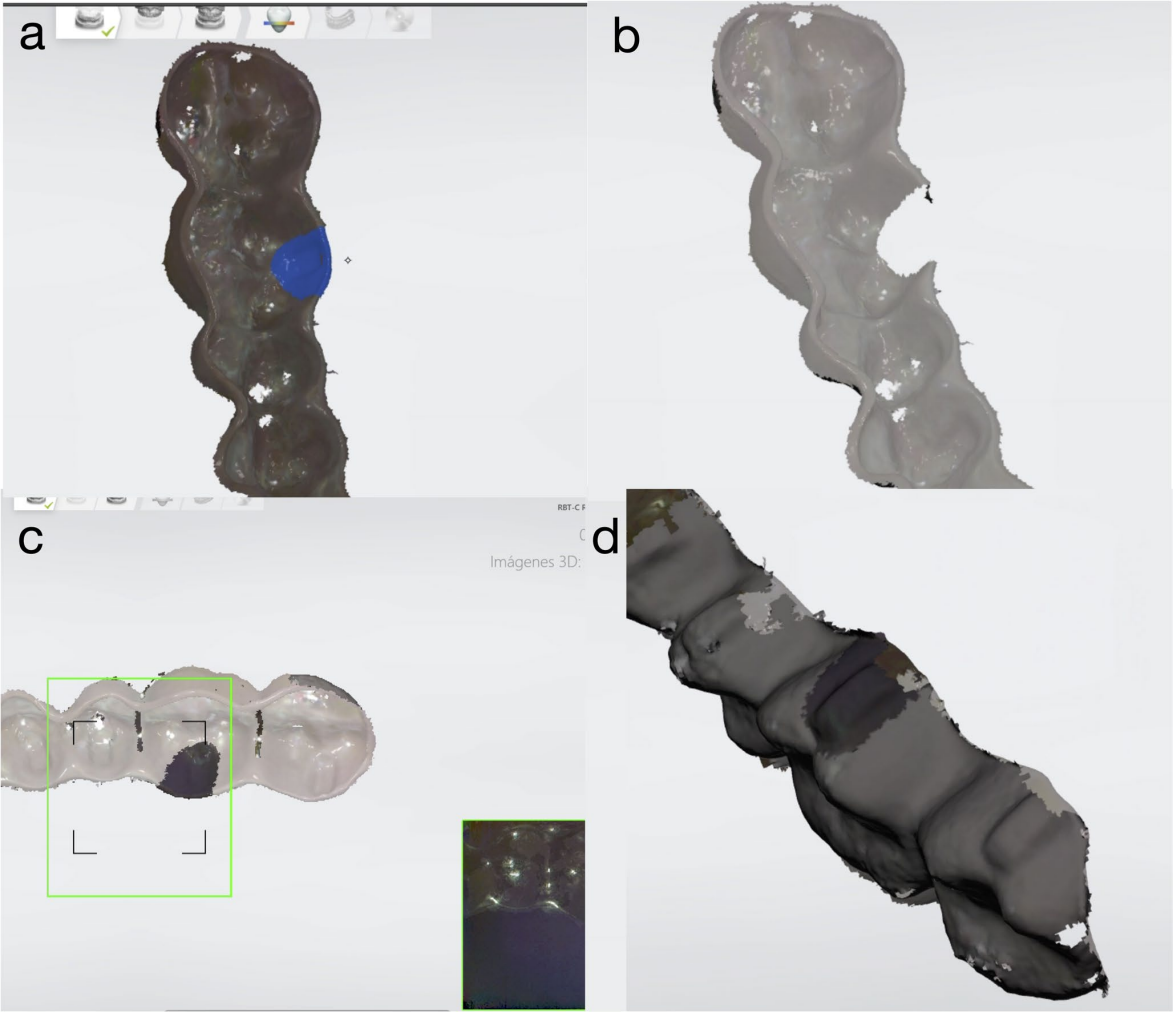
Processing steps for acquisition of STLs. (**a**) Baseline reference aligner STL with selection in color blue of Region of interest (ROI). (**b**) ROI mesh erased form STL. (**c**) Process of scanning of ROI of aligner after insertion/disinsertion test. (**d**) New STL file of reference aligner with new ROI

At each observation time, the aligners were scanned using a Trios 3 intraoral scanner (3Shape A/S, Copenhagen, Denmark). The external surface of the aligner was dyed with a black acrylic spray (Ref 703767 Nesboli Gravogroup^®^) to ensure accurate optic scanning of the region of interest. The region of interest (ROI) that would be assessed for volumetric changes was the attachment area of the first left molar. The first STL model was taken in T0 before any insertion/disinsertion cycles and was used as the baseline reference aligner, then this STL file was duplicated. Secondly, the duplicate file was selected, and the scan mesh of the region of interest (ROI) was erased. After, we scanned the ROI of the aligner subjected to the insertion/disinsertion cycles, filling in the previously erased mesh of the duplicate reference aligner. This process was repeated with each of the aligners at each observation time. Therefore, a total of 4 STL files were obtained for each aligner with a common general mesh (reference aligner), but with the mesh corresponding to the attachment region unique for each observation time. (Fig. 3)

**Fig. 4:**
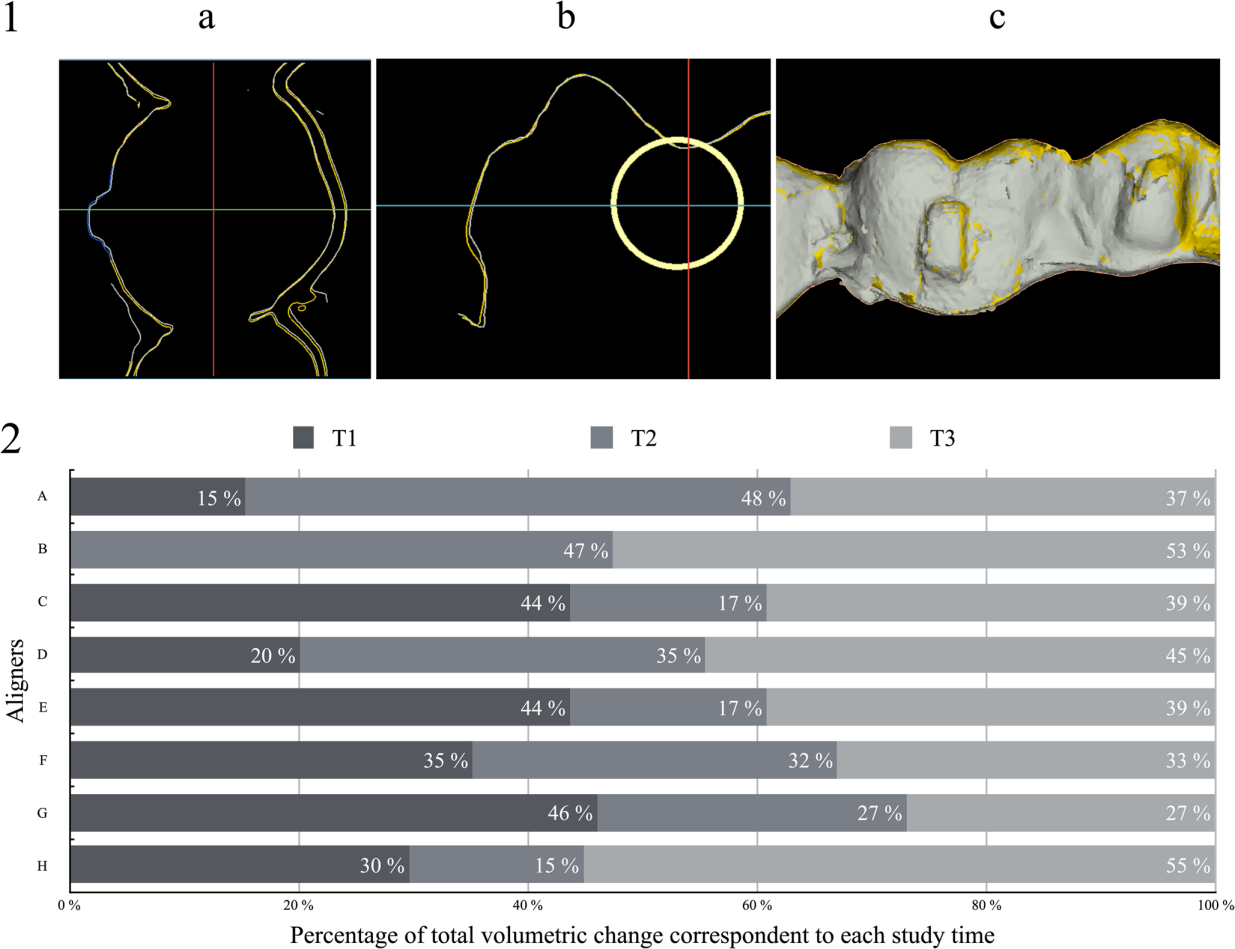
Volumetric change results. (4.1.**a**) Volumetric change comparison with SMOP software, axial section, blue area represents study area. (4.1.**b**): Volumetric change comparison with SMOP software, sagittal Sect. (4.1.**c**): Volumetric change comparison with SMOP software, superimposition of models. Figure 4.2: Percentage of total volumetric change corresponding to each study time of each aligner

Once the aligners had been scanned, superimpositions were made to observe the changes on the inner surface of the aligners in contact with the upper left first molar attachment. The volumetric change in the aligner for each observation time was measured by superimposing each STL file with the reference aligner STL (T0) (SMOP software, Swissmeda, Switzerland). The STL file corresponding to the reference aligner was imported into SMOP software to superimpose the rest of the STL files at different times over this one. The software performed an automatic overlay of the STLs based on the coordinate axes of each of the scans. These superimpositions were manually adjusted when needed. The volumetric change analysis region defined in the program (ROI) was a cylindrical surface with a radius of 2.5 mm enclosing the entire surface of the attachment. (Fig. 4−4.1)

### Statistics

The volumetric changes of the four groups of aligners at the different study times were evaluated using the mixed repeated measures ANOVA statistic. Related-Samples Friedman’s Two-Way Analysis of Variance by Ranks was applied to the variables that would not meet normality. In addition, the disinsertion force and disinsertion time would be evaluated using the same statistic. The statistical significance level was set at a p-value of less than 0.05 (*p* < 0.05). Additionally, the intraclass correlation coefficient (ICC) was calculated to evaluate the reliability of the measuring method with measured data within the same model. Data analysis was calculated using SPSS version 24 (SPSS Inc., Chicago, IL, USA).

## Results

### Maximum/mean disinsertion force considering aligner material, exposure to SIE, and level of crowding

**Fig. 5:**
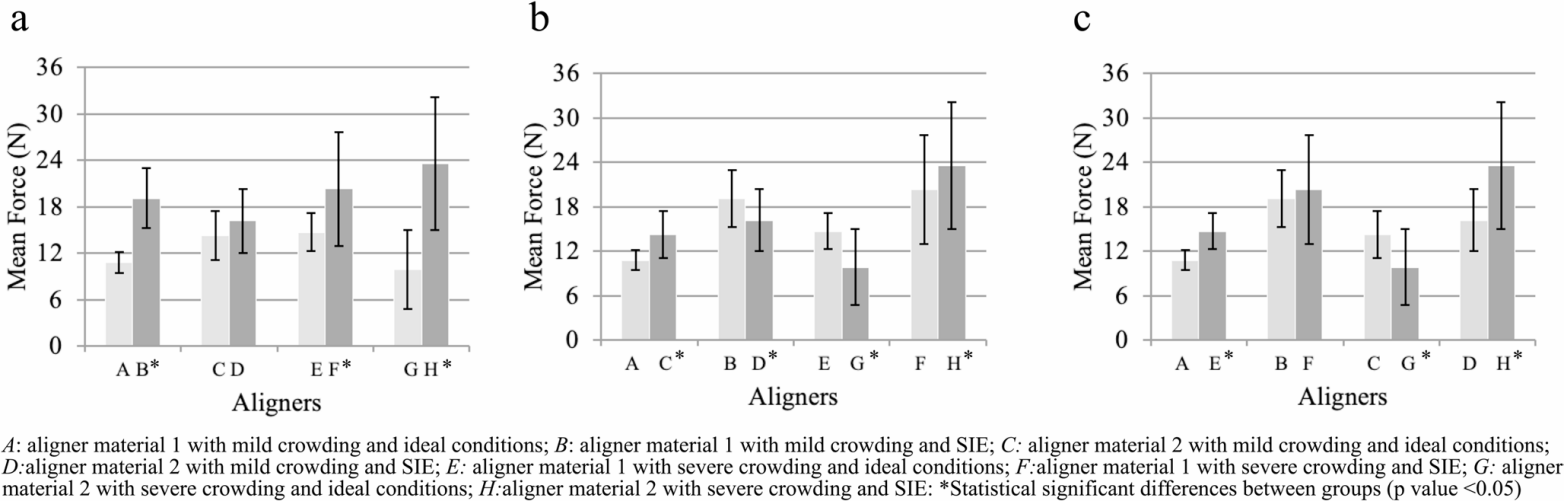
(**a**) Intergroup comparison of mean force SIE vs. ideal conditions; (**b**) Intergroup comparison of mean force aligner material 1 vs. aligner material 2; (**c**) Intergroup comparison of mean force mild crowding vs. severe crowding

The method retrieved optimum ICC results for all the tested experiments (ICC: 0.991). The results showed that the mean force of disinsertion of the Polyutherane-based (material 1) aligner samples significantly increased after immersion in SIE (*p* < 0,05), independent of the severity of the intraoral condition (mild/severe crowding). The specimens constructed with PET-G based polymer (material 2) and severe crowding also showed a significant mean increase in disinsertion force after immersion in SIE (*p* < 0,05). Additionally, the mean force of disinsertion tended to increase for the aligner material 2 specimen with mild crowding after immersion in SIE, but the difference was not statistically significant. (Fig. 5a)

Following immersion in SIE, the samples exhibited a mean rise in maximum disinsertion force of 56.2% (with a standard deviation of ± 26.25%) across 75 disinsertion cycles, in contrast to their performance under ideal conditions (Table 1).

**Table 1:**
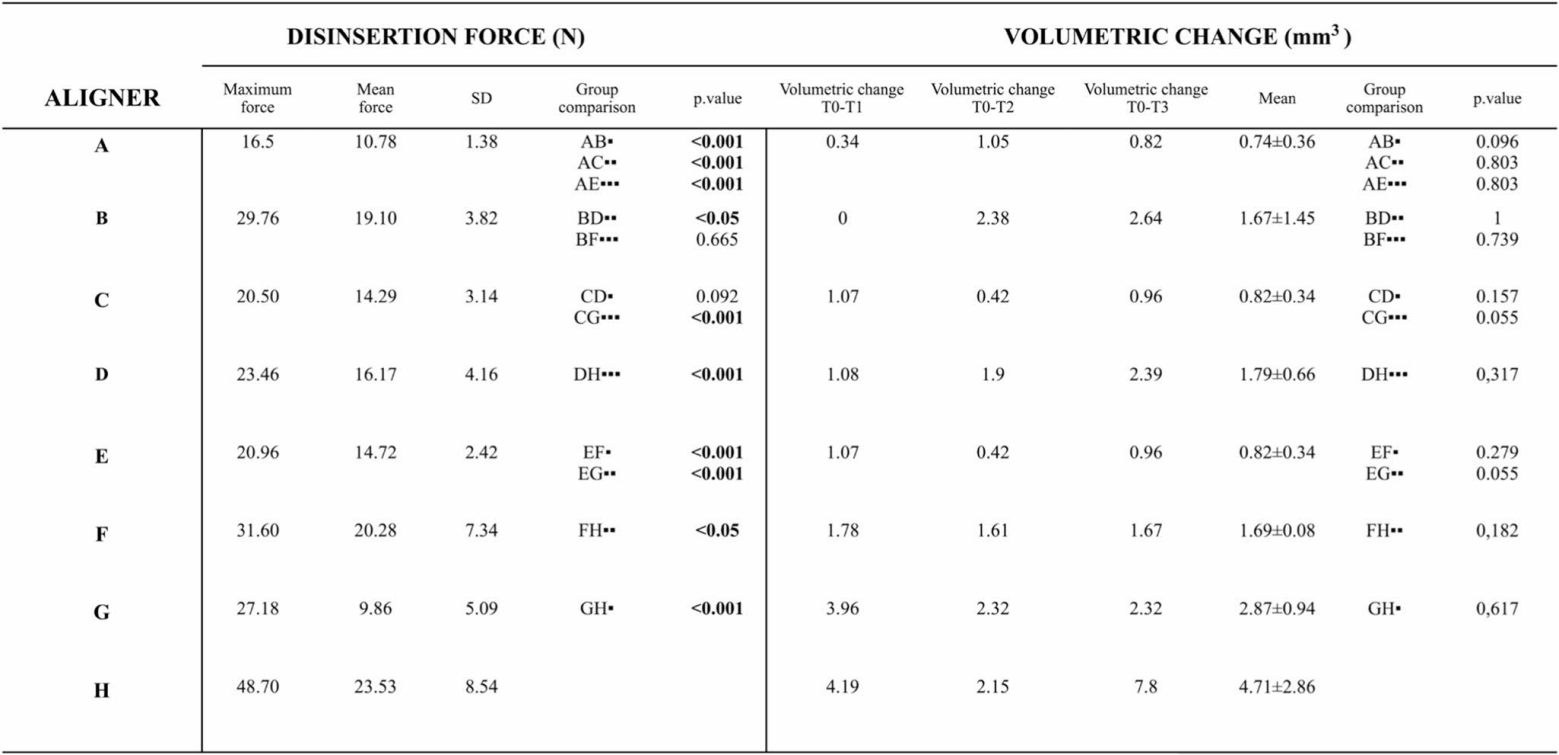
Results of cyclic insertion-disinsertion wear model on disinsertion force and volumetric change of materials

*A*: aligner material 1 with mild crowding and ideal conditions; *B*: aligner material 1 with mild crowding and SIE; *C*: aligner material 2 with mild crowding and ideal conditions; *D*: aligner material 2 wild mild crowding and SIE; *E*: aligner material 1 with severe crowding and ideal conditions; *F*: aligner material 1 with severe crowding and SIE; *G*: aligner material 2 with severe crowding and ideal conditions ; *H*: aligner material 2 with severe crowding and SIE. •Intergroup comparison SIE vs ideal conditions; ••Intergroup comparison aligned material 1 vs aligner material 2; •••Intergroup comparison mild crowding vs severe crowding

When comparing the effect of the aligner material on the maximum force of disinsertion in the samples under ideal conditions, the PET-G based polymer aligner samples showed an increase in the maximum force of disinsertion independent of the level of crowding compared to the Polyutherane-based aligner samples.

However, this tendency was not found when comparing the mean force of disinsertion. (Table 1) The sample from aligner material 1 with mild crowding and exposure to SIE exhibited a significant increase in the mean force of disinsertion compared to the sample from PET-G based polymer aligner material with mild crowding and SIE (*p* < 0.05). (Fig. 5b)

A significant increase in the mean disinsertion force was observed in the Polyutherane-based polymer aligner sample with severe crowding exposed to SIE compared to the same aligner with mild crowding (*p* < 0,05). The same tendency was observed in the Polyutherane-based aligner material 1 sample with severe crowding and ideal conditions. (Table 1) (Fig. 5c) Additionally, PET-G based polymer aligner samples manifested an increase in the maximum force of disinsertion when the crowding was severe.

### Deformation (volumetric change; mm3) of the attachment region considering aligner material, exposure to SIE, and level of crowding

The volumetric change was registered in each study time (T1-T3) and the mean volumetric change was recorded for each aligner. (Table 1) Compared with those under ideal conditions, the volumetric changes of all aligners submerged in the SIE tended to increase. This tendency of volumetric change was observed from the first study time (T1).

When comparing aligner materials, the PET-G based polymer aligner material demonstrated a consistently higher tendency towards volumetric change than Polyutherane-based polymer aligner material across all samples, with a mean increase of 132% (Table 1).

Additionally, the volumetric change depended on the level of crowding, with a greater volumetric change in the aligners with severe crowding. (Table 1) A greater percentage of the total volumetric change occurred in T1 in the aligners with severe crowding compared to the aligners with mild crowding. Additionally, aligners submerged in SIE showed a lower percentage of total volumetric change happening in T1 than those under ideal conditions. Finally, a greater percentage of the change was observed in T2 and T3 for the aligners submerged in SIE. (Fig. 4.2)

## Discussion

The present in vitro study aimed to assess the mechanical changes in two different aligner materials, in terms of the force of disinsertion of the aligner and volumetric change of the attachment region at three different study times (7,10 and 15 days), as well as the influence of SIE. This study revealed a significant increase in the mean and maximum force of disinsertion of the aligners after SIE exposure. Additionally, the deformation of the attachment region of all aligners submerged in SIE tended to increase compared to those under ideal conditions. Thus, inferring that the current materials used in the clinical setting are notably perturbed by the intraoral media, even in standardized non-hostile saliva media, due to the phenomenon of water absorption by the polymers, which modifies their biochemical structure [21, 22]. During this process, the hydrogen of the water molecules can interact with the polymer structure. However, most of the water remains in its free form within the polymer. The diffusion of the water in the organic matrix causes hygroscopic expansion, which may explain an increase in the deformation of the attachment region. This phenomenon of water absorption by polymers has previously been investigated in different thermoplastic aligner materials and polymeric restorative dental materials [16, 21, 23, 24]. The exposure to SIE showed a change in thickness and weight in samples of Ethylene–vinyl acetate copolymer (EVA), Polyethylene (PE), Polyethylene terephthalate glycol (PET-G), Polypropylene (PP), Polycarbonate (PC), Copolyester (A+), Polypropylene/ethylene copolymer (C+) and Polyurethane from methylene diphenyl diisocyanate (PUR). The absorption effect is more significant in amorphous plastics due to an increased free volume between the polymeric chains. Other properties, such as elastic moduli have been found to significantly increase in PET-G, PC, and A + after water absorption phenomena, increasing the stiffness of the material [16]. Importantly, this phenomenon is also considered to affect the hardness of polymers. Retrieved dental aligners (after 2 weeks of intraoral use) and dental aligners exposed to SIE, show increased hardness test results when tested with Vicker’s test [18]. Moreover, when specimens exposed to SIE are compared with new aligners, the indentation strength tests reveal an increase in hardness after intraoral use [15]. Increased crystallinity is observed after intraoral aging which has been associated with greater stiffness and less flexibility. Importantly, these findings might be related to our study, as a greater material hardness and stiffness may explain an increase in the retention of the aligner with the printed model. We observed an increase in the maximum and mean disinsertion forces of the aligners irrespective of the type of material when exposed to SIE, but more dramatically increased in the aligner material 2 samples. The difference between the results of both materials tested may be explained due to the different polymeric structures, blending processes, and additives incorporated in each material tested [9, 25]. Additionally, multilayer hybrid materials are being tested to enhance performance [26]. Aligner material 1 seems to have been engineered with additives to enhance water absorption resistance [27].

Additionally, the properties of dental aligner materials may alter with mechanical wear models [28]. The behavior of aligner materials subjected to a constant load for 24 h under SIE revealed a notable decline in stress across all tested materials over a 24-hour period. Initially, higher stress was observed within the first 8 h post-loading, followed by a subsequent decrease leading to a stabilized plateau. This suggests relaxation of the material and less stiffness. However, this result was taken only after 24-hour use [10]. Our study revealed that the mean force of disinsertion of neither of the materials was dependent on the number of disinsertion cycles. These results, show the significance of investigating the intraoral aging process of orthodontic materials to understand the mechanical properties of different aligner polymers. A multitude of potential aging factors, such as enzymes present in saliva, ph changes, and consumption of acidic beverages may play a role in the aging process [29]. However, most studies only use distilled water [10, 16, 22, 30]. Also, it is essential to consider individual factors associated with masticatory activity in intraoral retrieved aligners, explaining why results may differ among articles [15, 18, 31, 32].

Due to the multifactorial process of structural changes of the polymers, some studies have observed a decrease in hardness in retrieved aligners (44 ± 15 days) probably due to constant forces of mastication developed by the opposite dentition, therefore modifying the structure of the aligner [17]. Other studies, have also observed optical, chemical, and morphological changes in retrieved intraorally used aligners [18, 20, 32]. These studies used scanning electron microscopy and energy-dispersive X-ray microanalysis to find structural changes, microcracks, and biofilm depositions in the aligner materials [17, 33].

Aligner’s design and structure might also influence the pattern and magnitude of disinsertion force. Previous articles suggest that aligner disinsertion force is dependent on attachment morphology and placement [19–22]. The present study revealed a similar trend, with an increase in the force of disinsertion in those aligners with severe crowding, confirming that anatomical features and structural design of the aligners critically affect the force of disinsertion. The effect of the thermoforming process of the aligners has also been studied to modify the polymeric structure of materials [34]. To this respect, it has been described that the force and elastic modulus of PET-G and copolyester materials decreased after undergoing a thermoforming process. These findings indicate that the mechanical characteristics of thermoplastic materials for aligners need to be examined post-thermoforming to assess their suitability for clinical use.

Our study analyzed the properties of the aligner materials at three different study times (7,10 and 15 days). These study times were based on the suggested frequency different aligner brands recommend patients to change their aligners [5]. Also, aligner change protocol is based on complexity of tooth movement and collaboration of patients. Understanding the properties and behavior of different aligner materials at various study times can enhance the predictability of tooth movement. This allows for the establishment of an aligner change protocol for patients that also considers material characteristics.

Therefore, to understand the mechanical properties of the materials and from a clinical point of view, intraoral aging (masticatory activity enzymatic), aligner design, and the process of thermoforming should be taken into consideration when selecting the materials for dental aligners. However, this may pose challenges in research, as aligner-shaped testing samples introduce more intricate structures for analysis with conventional material testing equipment [15, 17, 34–36]. To overcome this limitation, there is variation in the samples tested by different studies, including non-teeth-shaped samples cut from non-thermoformed aligner sheets [16] or non-teeth-shaped samples from thermoformed sheets [18, 35]. This variability complicates the standardization of sample testing procedures.

When assessing aligner fit and volumetric changes, many studies have employed electronic microscopy. Initially, the aligner is fitted on a model and sectioned buccolingually with a cutting machine (Well Diamond Wire Saw Inc., Norcross, GA, USA). This sectioning is parallel to the long axis of the teeth and passes through the attachment region. The sample is then analyzed with an electronic microscope, and linear measurements of different reference points are taken [12, 37–39] Another method involves a micro-CT scan to measure the distance between the aligner and the model [8, 40]. However, most studies don’t analyze the fit and thickness after a cyclic wear protocol [34]. In our study, both aligner materials exposed to SIE tended to increase their volumetric change compared to ideal conditions. These results could have been due to the higher stiffness of aligners exposed to SIE. Moreover, an increase in the water absorption phenomena dependent on time may explain why aligners submerged in SIE showed a lower percentage of total volumetric change occurring in T1 and a higher percentage of the change happening in T2 and T3 [16].

The present research introduced and validated a novel method to evaluate the mechanical properties of aligner materials (force of disinsertion of the aligner from a printed model) and deformation of the attachment region (volumetric change) measured from an STL file. Despite the novel data provided within observed results regarding different intraoral conditions and media over different thermoforming materials, the current investigation exhibits certain limitations. It remains to be an in vitro analysis aimed at evaluating the performance of various aligner materials, without considering individual variables such as masticatory forces and more aggressive intraoral media, which in turn allow to prevent from heterogenous intraoral perturbances and guarantees reproducibility. Consequently, the experimental method used retrieved optimum ICC results as a proof of concept design. Critically, this method allowed direct assessment of aligners without having to section the samples for examination providing a comprehensive examination of the mechanical changes in aligner materials under simulated intraoral conditions. However, the process of defining the ROI presents certain limitations as we manually selected the center of ROI by visual assessment of the center of the attachment. Also, the alignment of the new scan of the ROI to the previous scan was automatically performed by the 3Shape scanner software. Therefore, the correct superimposition of both STL files may be affected by errors in manual selection and automatic alignment, leading to slight discrepancies in the analysis.

Future research should explore the behavior and properties of the aligners, both new and post-wear within the oral cavity and under hostile intraoral environment conditions. Beyond clinical performance, the use of polymeric materials raises important sustainability concerns. These polymers are derived from non-renewable resources and contribute to plastic waste, with limited options for recycling or biodegradation. Given the rapid expansion of aligner therapy, the cumulative environmental impact cannot be overlooked. Future research should explore eco-friendly polymers, improved recycling strategies, and circular production models to reduce the environmental burden of aligner therapy.

## Conclusion

1. The mechanical characteristics of thermoformed materials used for dental aligners undergo alterations due to exposure to SIE, observing an increased disinsertion force across all tested samples, likely resulting from an increased stiffness due to the phenomenon of water absorption. This effect can potentially affect the overall performance of aligners in clinical settings.
2. The progression of the deformation observed at the attachment region upon the volumetric changes is strongly affected by the material type and exposure to SIE. Aligners exposed to SIE tend to show increased volumetric changes, which are also time-dependent on the exposure to the medium. This might compromise the clinical behavior of the aligner.

3) Among the materials tested, the PET-G-based polymer exhibited a more pronounced tendency for volumetric changes compared to the polyurethane-based polymer across all samples. These differences highlight that aligner behavior is material-dependent. Therefore, the frequency of aligner replacement should not be standardized but instead defined according to the specific material properties to ensure optimal treatment efficiency and stability. However, these findings should be interpreted within the inherent limitations of an in vitro model and cannot be directly translated to clinical performance without supporting in vivo studies.

## Supplementary Information

Below is the link to the electronic supplementary material.

Supplementary Material 1 (MP4 26.5 MB)

## Data Availability

No datasets were generated or analysed during the current study.
